# Patch-augmented rotator cuff surgery (PARCS) study—protocol for a feasibility study

**DOI:** 10.1186/s40814-018-0380-7

**Published:** 2018-12-21

**Authors:** Jonathan A. Cook, Naomi Merritt, Jonathan L. Rees, Joanna C. Crocker, Sally Hopewell, Melina Dritsaki, David J. Beard, Amar Rangan, Cushla Cooper, Lucksy Kottam, Dair Farrar-Hockley, Michael Thomas, Robert Earle, Andrew J. Carr

**Affiliations:** 1 Centre for Statistics in Medicine, Nuffield Department of Orthopaedics, Rheumatology and Musculoskeletal Sciences, University of Oxford, Oxford, UK; 2 NIHR Oxford Biomedical Research Centre, Nuffield Department of Orthopaedics, Rheumatology and Musculoskeletal Sciences, University of Oxford, Oxford, UK; 3 Health Experiences Research Group, Nuffield Department of Primary Care Health Sciences, University of Oxford, Oxford, UK; 4 NIHR Oxford Biomedical Research Centre, Nuffield Department of Primary Care Health Sciences, University of Oxford, Oxford, UK; 5 The James Cook University Hospital, South Tees Hospitals NHS Foundation Trust, Middlesbrough, UK; 6 Frimley Park Hospital, Frimley Park Hospital NHS Foundation Trust, Surrey, UK

**Keywords:** Feasibility study, Randomised trial, Rotator cuff tear, Shoulder surgery, Tissue scaffold, Surgical mesh, Dermal matrix

## Abstract

**Background:**

A rotator cuff tear is a common disabling shoulder problem. Symptoms include pain, weakness, lack of shoulder mobility and sleep disturbance. Many patients require surgery to repair the tear; however, there is a high failure rate. There is a pressing need to improve the outcome of rotator cuff surgery and the use of patch augmentation to provide support to the healing process and improve patient outcomes holds new promise. Patches have been made using different materials (e.g. human/animal skin or intestine tissue, and completely synthetic materials) and processes (e.g. woven or a mesh). However, clinical evidence on their use is limited. The aim of the patch-augmented rotator cuff surgery (PARCS) feasibility study is to determine, using a mixed method approach, the design of a definitive randomised trial assessing the effectiveness and cost-effectiveness of a patch to augment surgical repair of the rotator cuff that is both acceptable to stakeholders and feasible.

**Methods:**

The objectives of this six-stage mixed methods feasibility study are to determine current practice, evidence and views about patch use; achieve consensus on the design of a randomised trial to evaluate patch-augmented rotator cuff surgery; and assess the acceptability and feasibility of the proposed design. The six stages will involve a systematic review of clinical evidence, two surveys of surgeons, focus groups and interviews with stakeholders, a Delphi study and a consensus meeting. The various stakeholders (including patients, surgeons, and representatives from industry, the NHS and regulatory bodies) will be involved across the six stages.

**Discussion:**

The PARCS feasibility study will inform the feasibility and acceptability of a randomised trial of the effectiveness and cost-effectiveness of a patch-augmented rotator cuff surgery. Consensus opinion on the basic design of a randomised trial will be sought.

**Trial registration:**

Not applicable.

**Electronic supplementary material:**

The online version of this article (10.1186/s40814-018-0380-7) contains supplementary material, which is available to authorized users.

## Background

### Clinical problem

Rotator cuff conditions relate to the tendons and muscles surrounding the shoulder joint. They account for up to 70% of shoulder pain problems and are the third most prevalent musculoskeletal disorder after lower back and neck pain [1, 2]. A severe but common rotator cuff problem is a rotator cuff tendon tear. Symptoms include pain, weakness, lack of shoulder mobility and sleep disturbance. Initial management is conservative and includes rest with simple pain management through paracetamol and non-steroidal anti-inflammatory drugs, often followed by an injection of corticosteroid into the subacromial space between the acromion process of the shoulder blade and the rotator cuff tendons below [3]. Physiotherapy involving strengthening and stretching exercises may also be used [4]. Approximately 40% of patients will continue to experience pain despite conservative management [5, 6], and many will require surgery to repair a tear in the rotator cuff.

### Surgery for rotator cuff repair

Surgical repair of the rotator cuff seeks to attach the tendon to the bone to allow the tear to heal and improve patient outcomes. Around 9000 rotator cuff repairs are performed each year in the NHS in England, at a cost of £6628 per operation (£60 million per year), and this number is continuing to grow [2, 7]. There is substantial variation in surgical practice, which includes the type of surgery (open or arthroscopic), surgical techniques (for example the use of anchors and type of suture), and type and duration of ancillary conservative treatment (including corticosteroid injections, physiotherapy, rest, advice and analgesia) [8]. Rotator cuff surgery can have mixed outcomes for patients [2]. It has a high failure rate (25–50% [9–11] within 12 months) and is expensive, invasive and inconvenient to patients. Re-operation is also sometimes necessary. Although there are different views about the key drivers of the health outcome, a number of factors are consistently related to poor outcomes, particularly increasing age and increasing tear size [12]. Four of the top 10 treatment uncertainties for common shoulder problems from a priority setting partnership for surgery for common shoulder problems’ involving patients, carers and clinicians [13] concerned rotator cuff tears. There is, therefore, a pressing need to progress surgical options for rotator cuff repairs and to improve tendon healing and outcomes for patients [14].

A number of unsuccessful surgical approaches have been tried to improve the outcome of rotator cuff repair [2, 11, 15, 16]. The United Kingdom Rotator Cuff Trial (UKUFF) trial found that minimally invasive (arthroscopic) surgery had no extra benefit over open surgery [17]. An updated systematic search revealed six more trials comparing two surgical interventions [18–23]. These RCTs were single centre and were relatively small and mainly included participants with full thickness rotator cuff tears and with small and medium rotator cuff tears. One further ongoing study was identified [24]. There was no evidence that the use of suture anchors or alternate methods of suturing improve healing rates. Attention has recently focused on improving the biology of the torn tendon at the time of surgery and for the critical 8–12-week period after surgery, when effective healing is needed [25]. Repairs commonly fail due to poor tissue and bone quality or inadequate fixing of the tendon to the bone, allowing the tendon to pull away from the bone.

### Patch-augmented rotator cuff surgery

A promising, under-evaluated area for further assessment is the use of a patch to provide a support structure or ‘scaffold’ for the repair, to improve the fixing of the tendon to the bone and tendon healing [26, 27]. These implants are also referred to as an extracellular or acellular matrix (when made from human or animal cells) or as a graft (e.g. an allograft, autograft or xenograft, depending on the source material used to manufacture the patch). The patch is surgically sutured on top of the tendon-to-bone repair to strengthen the repair and aid tendon healing, thereby reducing the likelihood of failure and improving patient outcomes. [28]

Patches have been made using different materials (human/animal heart, skin or intestine tissue, and completely synthetic materials) and processes (e.g. woven or mesh approaches) and to different sizes. They can be designed to be absorbable, avoiding the possibility of later surgical complications or surgical removal. [29] Patches differ in how they respond to tendon tissue and their mechanical properties. [30] Some have been designed specifically or can be tailored in size and shape for specific use in rotator cuff surgery, whereas others have been developed for other soft-tissue contexts (e.g. anterior cruciate ligament reconstruction in the knee or for hernia repair). Recent advances include the development of electrospun materials [31] and exploration of the concurrent use of growth factors. Electrospun materials have a structure that closely resembles the surrounding tissue; they provide biological cues to encourage cell growth and tissue healing. The aim of these and other biomimetic materials is to avoid adverse immunological responses, which some tissue-based patches have provoked [32]. Augmenting surgical repair with a patch may also enable the repair of tears that are currently considered unrepairable [26, 29, 31, 33, 34]. A number of patches have received regulatory approval in the USA and/or by an EU-notified body for use in surgical repair of the rotator cuff. There are more patches in development. There is currently a window of opportunity to design, gain stakeholder buy-in for, and conduct a timely randomised controlled trial (RCT) before widespread adoption of these medical devices for rotator cuff surgery. However, the design and feasibility of such a trial is not clear. Key uncertainties about the design and conduct of such a trial include the types of patches that are in clinical use in the NHS, which should be evaluated in a trial, which patients would benefit most, how the surgery should be delivered and which outcomes should be measured.

## Study design

### Aim and objectives

The aim of this study (PARCS) is to determine the design of a definitive randomised trial assessing the effectiveness and cost-effectiveness of a patch to augment surgical repair of the rotator cuff tendon that is both acceptable and feasible.

The specific objectives are to:

1. Review existing evidence to identify candidate patches for use in a randomised trial and the evidence relating to their clinical use,
2. Determine current practice in the NHS relating to the use of patches to augment rotator cuff repair,
3. Assess the acceptability of a trial assessing patch augmented rotator cuff repair to patients and surgeons,
4. Assess the feasibility of a trial of patch-augmented rotator cuff repair,
5. Achieve consensus on the key elements of the design of a definitive randomised trial to assess the use of patches to augment rotator cuff repair,
6. Confirm the scope of the health economic evaluation required in the trial to appropriately assess its cost-effectiveness,
7. Identify areas for further relevant research related to patch-augmented rotator cuff surgery.

**Fig. 1:**
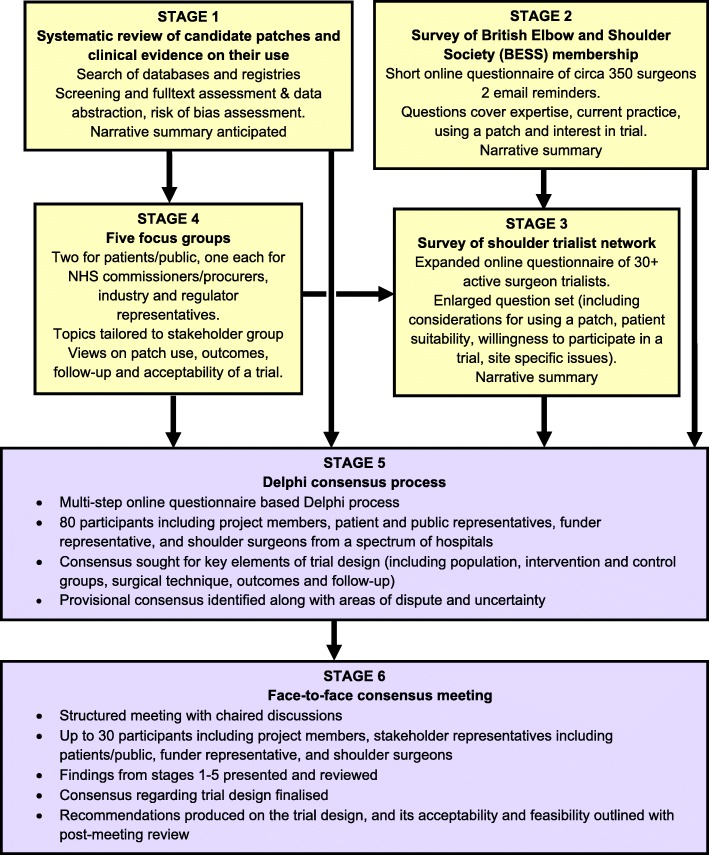
Study flow chart. Patch-augmented rotator cuff repair study (PARCS)

PARCS is a mixed methods feasibility study consisting of six stages. The design of each stage is given below. As this protocol pertains to stages 2–6 only, a very brief summary of stage 1 is provided. The consensus methods approach adopted builds on the work by the IDEAL Collaboration for evaluating surgical innovation and devices in early-stage and randomised trial assessments [35] and adapts the methodology used for achieving expert consensus in guideline development and development of core outcome sets [36–38] to the broader scope of trial design, see Fig. 1 for a summary flow diagram.

### Stage 1: Systematic review of candidate patches and related clinical evidence

A systematic review of the surgical management of rotator cuff repair with augmentative patch will be performed. This protocol is available elsewhere [39].

### Stages 2 and 3: Surveys of surgical practice, acceptability and feasibility of a randomised trial

The main objective of the two surveys together is to ascertain current NHS clinical practice relating to the use of patches to augment rotator cuff repair and practical issues related to conduct of a trial in this area.

#### British Elbow and Shoulder Society (BESS) membership survey (stage 2)

The aim of this survey is to identify current UK clinical practice and gather information on surgeon opinion relating to the factors that influence their choice of patch and patient suitability. This survey will also explore the interest in participating in a RCT of patch augmented rotator cuff repair. An invite to participate in this online survey will be sent to all surgeon members of the British Elbow and Shoulder Society (BESS). The main mechanism of approaching potential participants will be via the BESS society email list; the invite will be sent out by the BESS office to avoid unnecessary sharing of personal data with the PARCS team. Information about the study and a hyperlink to the relevant survey will be provided. Reminder emails will be sent to the entire sample in a similar manner. The survey will be delivered online using the Bristol Online Survey (BOS) tool or an equivalent system [40]. Surgeon members of BESS attending the 2017 annual meeting will be offered an opportunity to complete the survey during the meeting. A member of the PARCS study team will provide verbal and, where appropriate, written study information. If the surgeon is agreeable, they will be given access to the online survey for completion at the meeting. Prior to finalising, the survey will be piloted internally amongst the study investigators and a number of external individuals as appropriate. The survey is anticipated to take approximately 10 min to complete and is available in Additional file 1. The BESS membership includes approximately 350 clinically active shoulder surgeons. There is no minimum number of responses required. The response rate will be defined as the number of responding participants divided by the number of eligible people invited. The statistical analysis will be descriptive only. Responses will be summarised quantitatively or narratively, as appropriate (e.g. using Microsoft Excel and/or Stata).

#### Survey of shoulder surgeon trialists (stage 3)

The second survey will build upon the findings of the first survey and will be for a subset of surgeons who have previously been actively involved in previous UK shoulder surgery trials as study investigators. The surveys will also partly address trial acceptability and feasibility (patient population, surgical procedures and practical recruitment process considerations) from the surgeon perspective. It will be directed at surgeons who are trial active and, therefore, most likely to participate in a randomised controlled trial of patch-augmented rotator cuff surgery. A network of surgeon trialists who have participated in previous NHS-based shoulder surgical trials will be invited to take part. This will include surgeons who acted as principal investigator for the UKUFF, CSAW and UKFroST trials [2, 41, 42]. At least 30 research active orthopaedic shoulder surgeons will be invited to take part. This is considered large enough to meet the aim of this component of the project and ensure that a range of surgeons and centres are included. To be eligible, an individual will need to be a practicing orthopaedic shoulder surgeon who is currently, or has previously been, an investigator for a RCT of shoulder surgery, i.e. experienced in recruitment, trial treatments and completion of case report forms. Eligible participants will be identified by the PARCS Study Management Group based upon the advice of the trial managers of the aforementioned shoulder surgery trials. The PARCS Study Management Group includes individuals who were involved in all of these trials. They will be recruited through a personalised, email or face-to-face invitation. These surgeons will be invited based on their previous experience in shoulder surgical trials. Email correspondence (invites/reminders where needed) will be personalised. During the survey, participants will be asked to register their interest in taking part in further stages of the PARCS study. The number of responses and feedback received on completing the stage 2 survey will be taken into account when finalising the stage 3 survey (see Additional file 2). This survey is anticipated to approximately 15 min to complete. There is no minimum number of responses required. The survey data will be analysed in a similar manner to the stage 3 survey.

### Stage 4: Focus groups

Focus groups allow participants to speak freely about their concerns and offer their views about the existing and proposed evaluation of a new approach to surgical treatment. They are particularly useful for helping to identify issues that resonate with lay people and the public at large in matters of healthcare [43, 44]. Focus groups are widely used in health services research.

Using a number of focus groups, we aim to access a broad range of stakeholder views and opinions on the acceptability of the use of patches in the augmentation of rotator cuff repair and the trial design options that may be used to test them. Themes and issues identified from the surgeon survey (stages 2 and 3) will help to form topics for discussion if findings are available when this stage is ongoing.

Members will be recruited to separate focus groups, each reflecting the various key stakeholders:

A. Patients/public (including carers) with current or previous rotator cuff or other shoulder problems. This included but was not limited to those who had undergone rotator cuff surgery
  Two focus groups will be conducted, one each in two regions of England (Thames Valley in the south and Tees Valley in the north)
B. Representatives from Industry
C. Other stakeholder representatives, including regulatory bodies, commissioners and the NHS such as those involved in research delivery and procurement of surgical equipment

Group A is considered to be the key stakeholder group. However, the introduction of patches into the NHS has regulatory and cost implications; therefore, it is relevant to include the views and opinions of groups B and C in the study. Potential participants will be invited and recruited using various avenues.

Consultant orthopaedic surgeons (and PARCS Study Management Group members) based at the Nuffield Orthopaedic Centre, Oxford and the James Cook University Hospital, South Tees, will approach potential patient participants though outpatient and physiotherapy clinics. Study staff may also contact patients previously known to study clinical investigators at the two sites. If patient/public uptake is low, poster advertisement in the local community may also be used, and if necessary, the geographical area extended. Local or national websites set up to increase public and patient involvement in clinical research may also be utilised. Other stakeholders and individuals with relevant experience and knowledge will be identified, approached and invited to participate directly. This may be through professional or personal acquaintance. If necessary, the technique of snowballing may be utilised, i.e. respondents may be asked to pass on information to other potential participants [45]. A balance of men and women within each focus group will be aimed for.

Each focus group will aim to involve four to eight participants and will be held at the Botnar Research Centre, University of Oxford (Oxford, Thames Valley, UK), the South Tees Institute of Learning, Research and Innovation (Middlesbrough, Tees Valley, UK), or where possible, at a location better suited to participants (e.g. their place of work or by teleconference). Focus groups sessions will last for a maximum of 2 h. There will be breaks of at least 15 min per hour of discussion. Refreshments will be provided during the focus group session.

Focus groups will be facilitated by a trained member of the PARCS study team. Discussions will be audio recorded, and one or two observers will take notes to aid in the transcription of audio files and analysis. Participants will provide their written consent prior to audio recording. Any identifying information appearing in focus group transcripts will be replaced with a pseudonym as soon as possible following transcription to minimise risk of participant identification.

Ahead of the focus group session, potential participants will be provided with a study information sheet (specifically tailored to their stakeholder group) that describes the aim of the focus group, what taking part will involve and the consent procedure. Depending on individual preference, this information will be supplied by hand, email or post. Written informed consent will be obtained. As soon as they are confirmed, arrangements for the focus group (date, time and location) will be provided.

During the focus group session, the aim of the focus group and the PARCS project will be briefly introduced and participants will be asked to consider a number of relevant issues, scenarios or vignettes. Key items of information about the possible trial design options, such as the different kinds of patches available, the choice of study arms, most appropriate outcome measures and methods of data collection (e.g. biopsy, patient questionnaires, site visits), will be raised. The way in which this information is delivered, and the level of detail considered, will likely be adapted according to participant group.

Participants of the patient/public focus groups will be asked to provide some basic background information about themselves (gender, age, relevant experience and previous treatments). To ensure anonymity, participants will be provided with a plain opaque envelope in which to place the completed ‘background information form’ and will be instructed to place the envelope in a box as they leave.

If it is not feasible to conduct a focus group meeting, or for all individuals to make the same date and location, potential participants may be offered an individual or, as a subset of all participants, a separate face-to-face or telephone interview instead. Participants will have the right to leave requests for information unanswered if they wish. We will reassure these participants and inform them that there will be no adverse consequences from this and are free to withdraw themselves at any time.

Data collected at the focus groups (stage 4) will be analysed alongside data collection using thematic analysis [46]. The emphasis of the analysis will be on the acceptability of the proposed trial and on factors that might facilitate or impede such acceptability. Thematic content analysis will be carried out by three members of the PARCS study team (CC, NM JCC) and will consist of the following steps:

1. Familiarisation with the focus group transcript,
2. Coding the transcript text under relevant themes,
3. Agreeing a thematic framework,
4. Applying the framework to subsequent focus group transcripts,
5. Interpreting and summarising the data within each theme,
6. Drawing out implications for trial design and stages 5 to 6 of the feasibility study (JAC will also be involved in this step).

Coding will be both deductive (guided by the themes included in the focus group topic guides) and inductive (allowing the emergence of unanticipated themes and sub-themes). Steps 3 and 4 will be iterative, i.e. the thematic framework may be refined or modified and reapplied to transcripts as the analysis progresses. Changes to the thematic framework will require agreement of all members involved in steps 1–5.

### Stage 5: Delphi study

A Delphi study to develop a consensus on the best way to design a clinical trial of patch-augmented rotator cuff surgery will be conducted. The Delphi method is a structured process of obtaining information from a group of experts using a series of related questionnaires, each one refined using respondents’ feedback from a previous version [47]. Delphi is a well-known and increasingly common method used in the clinical setting to establish a consensus [43, 47, 48]. A multi-stage online Delphi survey consisting of at least two but no more than three rounds will be conducted. The survey will be developed and conducted using the BOS System or an equivalent [40].

Participants involved in stages 2 to 4 of the PARCS Study will be invited to take part in stage 5 according to stakeholder group and background. Given the nature of the study, there has been no formal sample size calculation but around 50–80 are anticipated. There are generally no accepted guidelines for the optimal sample size needed to achieve consensus in a Delphi studies [49]. This sample size was based on previous experience of conducting this type of survey and anticipated attrition rates at each round. Substantial loss from the initial to final round is not unusual [48, 50].

Where appropriate, stakeholders who have relevant experience but did not take part in previous stages may be invited to participate. These potential participants will be identified and recruited using a similar approach as described in stages 3 and 4. Electronic confirmation of consent will be obtained.

Delphi study participants will have their name and contact email address entered in to the suryey system [40]. An email will be sent to each participant containing a personalised link that enables access for survey completion. Findings from stages 1 to 4 will determine the design elements to be included in the first round of the Delphi survey. Two versions of the survey will be used one for patient and public stakeholders and one for professional (e.g. surgeons, researchers) stakeholders. The former version will have a subset of the full set of question which are most pertinent to this stakeholder group and will be presented using more accessible language and avoiding as far as possible technical terminology.

During completion of the first round, survey participants will be asked to supply some basic demographic information (for example, age, background, current employment and position (professionals only), relevant medical history (patients/public) and number of years of relevant experience) and will be allocated a unique identifier used for administrative and data analysis purposes. Participants will be presented in the survey with proposed elements (e.g. choice of two or three arm trial design, eligibility criteria for participation and information on the timing of the outcome data collection) of the trial design and asked to score agreement with each design element using a 1–5 scale, where 1 represents complete disagreement and 5 represents complete agreement. Participants will be given the opportunity to communicate their personal suggestions with regards to changes to a design element or any additional design elements they feel should be included in future rounds in order to achieve consensus. All initial design elements will be carried forward to subsequent  rounds of the Delphi survey though the content will be adaptable dependent upon the response received. New design elements suggested by participants in round one of the Delphi survey will be reviewed at least two members of the PARCS study team. The wider project team will be consulted if there is any uncertainty or to decide if an additional element should be added.

Participants will receive a summary of the findings from the previous round in subsequent rounds. They will be asked to reflect on their own responses and also the collated responses. Participants will then score each design element. The final set of proposals, areas of provisional consensus and remaining disagreement and uncertainty will then be brought forward to the consensus meeting in stage 6 and used as the basis for discussion.

Where necessary, at each round of this Delphi survey, non-responders will receive a maximum of two reminder messages. The final reminder will contain a specific deadline for survey closure [51]. Each survey will take approximately 20 min to complete.

Scores (range 1–5) from each round will be calculated as a percentage of the total responses. We will define consensus for the content of the design elements proposal as > 70% of responses rating the element 4 or greater and not more than 15% of responses rating the element 1. Median and ranges will also be produced for the scores. We will explore similarities and differences across stakeholder groups. Textual responses will be summarised narratively.

### Stage 6: Consensus meeting

Findings from stages 1–5 will feed into, and inform the structure of, a 2-day face-to-face meeting where the final consensus on an acceptable and feasible trial design for a definitive randomised trial to assess the effectiveness and cost-effectiveness of a patch to augment surgical repair of the rotator cuff tendon will be sought. This meeting will involve a range of stakeholders (including patient and public representatives, surgeons and trialists who took part in stages 2–5 of the study). Participants will be selected for invite based on their perspectives, experience and background. This will be done in order to ensure a range of stakeholder groups are represented and individuals from different backgrounds and experiences (e.g. surgeons who do currently use patches to augment rotator cuff repair along with those who would be potentially willing to do so for a trial). To ensure a robust decision is made, approximately 30 stakeholders will take part in this meeting.

Ahead of the consensus meeting, participants will be sent a summary of findings from earlier stages of the project. Patient and public representatives will be reimbursed for expenses and compensated for their time in line with current NIHR INVOLVE guidelines on payment and recognition for public involvement [52]. The meeting will be structured to ensure key areas of uncertainty and/or disagreement are identified. Consensus on key elements of the trial design: patient eligibility, intervention and control definitions, surgeon requirements, outcomes and target difference will be sought. Draft guidance, options and recommendations for a randomised trial assessing patch-augmented rotator cuff surgery will be developed from previous work updated in light of the findings from previous stages.

A post-meeting report will be drafted and circulated to participants for their review and comments. The report will detail the key design decisions and will be divided into sections on methods, study design issues (e.g. the definition of comparison groups) and special topics (e.g. allowable variation in surgical technique).

### Project management and adherence to regulatory requirements

The investigators will ensure that this study is conducted in accordance with relevant regulations and with the Good Clinical Practice. The study will be conducted in accordance with the current approved protocol, relevant regulations and standard operating procedures. The independent Study Steering Committee (SSC) will oversee study conduct and progress. A potential participant will be allowed as much time as they wish (within the constraints of the project timelines) to consider the information, and the opportunity to ask questions. At all stages of the study, it will be clearly stated that a participant is free to withdraw themselves and/or their response data at any time where it can be identified and removed (this is not likely to be feasible for responses to stages 4–6). There will be no adverse consequences or impacts on future care if this is done.

The study will comply with the Data Protection Act, which requires data to be anonymised as soon as it is practical to do so. Following publication of our findings, anonymised individual participant data (as far as is feasible according to the nature of the data) will be permanently archived. Anonymised data may be shared with legitimate internal and external researchers. The chief investigator will act as the data custodian for this study.

The investigators will also ensure that this study is conducted in accordance with the principles of the Declaration of Helsinki.

### Progress to date

The stage 2 and 3 surveys have now been completed, and an initial summary of the findings produced. Focus groups and interviews (stage 4) have now also been completed, and a summary of findings of this stage produced. Stage 5 is in progress with a date set for the consensus meeting (stage 6) in early 2019.

## Discussion

Rotator cuff tears are a relatively common problem, and the number of operations to repair them is likely to grow in developed countries over the foreseeable future as the population ages. Despite benefits for many patients, the operation does not provide sustained benefit for a substantial minority. The augmentation of the operation with a patch seems promising with some evidence of clinical benefit. However, which patients might benefit most and the extent of such a benefit is unclear particularly in what might be described as a typical patient. Furthermore, there is key uncertainty regarding the available patches, the clinical evidence on their use and the views of key stakeholders particularly patients and surgeons on their use. When the prospect of conducting a large multicentre randomised trial to evaluate their use is considered, it is clear that a preparatory research would be highly valuable to inform the design of such a study and indeed if it is feasible to conduct. Furthermore, various trial design options are possible and it is not clear a priori which is most appropriate.

The main strengths of the study design are the planned systematic involvement of a variety of different stakeholder groups and the use of multiple quantitative and qualitative methodologies, in order to seek to produce the most informed output from the study. The main weaknesses of the study are the relatively slow and more time-consuming nature of the overall study compared to simpler feasibility study designs. Participants in stages 2–6 may not be fully representative of all stakeholders or reflect the full range of viewpoints and perspective. For example, surgeons who use patches in their clinical practice may have been more likely to participate in the stage 2 and 3 surveys. This could limit the generalizability and applicability of the findings.

The PARCS feasibility study seeks to address this gap in knowledge and seeks to take an inclusive approach with a variety of research methodologies utilised to inform the feasibility and acceptability of a randomised trial in this area. Specifically, one that would evaluate the effectiveness and cost-effectiveness in the context of the NHS in the UK is in mind. It is hoped that this study might lead to the funding and conduct of such a study.

## Additional files

Additional file 1: PARCS BESS Membership Survey. (PDF 156 kb)

Additional file 2: PARCS Surgeon Trialists Survey. (PDF 102 kb)

## Abbreviations

BESS: British Elbow and Shoulder Society
BOS: Bristol Online Survey
CSAW: Can Shoulder Arthroscopy Work?
EU: Europe/European
HRA: Health Research Authority
IDEAL: Idea, Development, Exploration, Assessment, Long-term follow-up
MHRA: Medicines and Healthcare products Regulatory Authority
NHS: National Health Service
RCT: Randomised controlled trial
SSC: Study Steering Committee
Stata: Data Analysis and Statistical Software
UK: United Kingdom
UKFRoST: United Kingdom Frozen Shoulder Trial
UKUFF: United Kingdom Rotator Cuff Trial
USA: United States of America
