# Supplementary material for: Patch-augmented rotator cuff surgery (PARCS) study—protocol for a feasibility study
Source: Pilot Feasibility Stud. 2018 Dec 21;4:188. doi: 10.1186/s40814-018-0380-7 (PMC6302398; doi:10.1186/s40814-018-0380-7)
Supplement: Supplementary file 2 — PARCS Surgeon Trialists Survey. (PDF 102 kb) [file 40814_2018_380_MOESM2_ESM.pdf]

PARCS Surgeon Triallist Survey

Introduction

Dear Surgeon Colleagues

Currently we are developing a trial looking at the use of patches for rotator cuff tears in the NHS setting. This work is within a project called PARCS (Patch Augmented Rotator Cuff Surgery). Professors Andy Carr, Amar Rangan, Jonathan Rees and Mr Mike Thomas, along with Professor Jonathan Cook are involved in the study, amongst others.

You may have answered a survey related to this earlier last year. As a further step in designing a rotator cuff patch trial, we are asking you to complete a survey related to how such a trial could be designed.

This survey should take about 10 minutes to complete.

Please note: this survey needs to be completed in one-sitting to avoid losing data.

All data analysed will be anonymous. However, we do request your contact details if you would like to be involved in future PARCS work (a Delphi study).

If you have any queries please contact Cushla Cooper at rcrstudies@ndorms.ox.ac.uk.

Many thanks from the PARCS team

The PARCS project is funded by National Institute for Health Research's Health Technology Assessment Programme (15/103/03).

https://www.ndorms.ox.ac.uk/clinical-trials/current-trials-and-studies/parcs

Patch use for rotator cuff repair

Do you currently use a patch to augment a rotator cuff repair on any of your patients?

If you do use a patch, how do you typically use it?

If you selected Other, please specify:

Would the state of the subscapularis muscle affect your decision to use a patch?

Please explain your answer:

A trial of patch augmented rotator cuff

Please consider which patients **you would be prepared to randomise** to each trial scenario.

Tick all that apply

(we realise not everyone within a category would be considered the same but we are interested in *which patient subgroups you would consider using a patch*)

Randomised Controlled Trial comparing a rotator cuff repair *plus a Patch* **versus** a standard cuff repair *with No-Patch* (2-arm study)

- ☐ Medium tear
- ☐ Large tear
- ☐ Massive tear
- ☐ Revision
- ☐ 50-60 years
- ☐ 60-70 years
- ☐ 70-80 years
- ☐ 80+
- ☐ Would not randomise any patients into such a study

Randomised Controlled Trial of standard rotator cuff repair *plus Patch A* **versus** standard rotator cuff repair *plus Patch B* **versus** standard rotator cuff repair *with No-Patch* (3-arm study)

- ☐ Medium tear
- ☐ Large tear
- ☐ Massive tear
- ☐ Revision
- ☐ 50-60 years

- [ ] 60-70 years
- [ ] 70-80 years
- [ ] 80+
- [ ] Would not randomise any patients into such a study

Please consider each patient characteristic below in turn, and confirm whether you think **any of the subgroups should be excluded** from a trial of patch augmented rotator cuff repair.

Tick all that apply

Degree of atrophy present

- [ ] Grade 0 - normal muscle
- [ ] Grade 1 - some fatty streaks
- [ ] Grade 2 - less than 50% fatty muscle atrophy
- [ ] Grade 3 - 50% fatty muscle atrophy
- [ ] Grade 4 - greater than 50% fatty muscle atrophy

Optional comments on degree of atrophy present:

Degree of glenohumeral osteoarthritis present (Kellgren Lawrence Classification)

- [ ] Grade 0 - No radiographic evidence of osteoarthritis
- [ ] Grade 1 - Marginal osteophytes of doubtful importance
- [ ] Grade 2 - Definite osteophytes
- [ ] Grade 3 - Moderate joint space narrowing, subchondral sclerosis
- [ ] Grade 4 - Severe joint space narrowing cyst formation

Optional comments on degree of glenohumeral osteoarthritis present:

Presence of cuff arthropathy:

- Yes
- No

Optional comments on cuff arthropathy:

Trial procedures

When to randomise

When would it be most practicable for a patient to be randomised to a treatment allocation?

- ◌ Prior to the day of surgery (eg: pre-surgery assessment)
- ◌ In the anaesthetic room
- ◌ In the operating room (at the start of the operation)
- ◌ In the operating room (once the shoulder pathology has been assessed)
- ◌ Other (please explain)

If you selected Other, please specify:

Consider what you would be willing to do in a trial.

For a trial, we would work with surgeons to agree a standardised repair technique suitable to the trial patient population. Within a trial, would you be willing to:

- ◌ Use an agreed standardised repair technique
- ◌ I would not want to use a standardise repair technique, I prefer to use my own repair technique

Optional comments on use of a standardised repair technique: *Optional*

Would you be willing to only use a specific patch? (i.e: a brand of patch, not a generic type)

- Yes
- No

Optional comments on using a specific patch within a trial:

Would you be able to use a standardised post-operative rehabilitation regime for patients in a trial?

- Yes
- No

Optional comments on standardised post-operation rehabilitation regime within a trial:

What length of patient follow up is requried post rotator cuff repair to assess the outcome of the operation?

- 6 months
- 12 months
- 24 months
- Other

If you selected Other, please specify:

Further contact

If you would be interested in being involved in the Delphi study seeking consensus on the key aspects of the design of the RCT on this topic, please enter the best email address to contact you about this below:

Thank you for completing this survey.

Any further comments

Thank you for completing this survey

Thank you for completing the PARCS Survey!

Key for selection options

1 - Do you currently use a patch to augment a rotator cuff repair on any of your patients?

Yes
No, but I would be willing to for a trial of patch augmentation with suitable support.
No, and I would not be interested in being involved in a trial where I would have to carry out patch augmentation.

2 - If you do use a patch, how do you typically use it?

Bridge (used to fill a persistent defect after a standard repair)
On-lay (used to reinforce a standard repair)
Other, please state

3 - Would the state of the subscapularis muscle affect your decision to use a patch?

Yes
No
